# Prospects for Moxidectin as a New Oral Treatment for Human Scabies

**DOI:** 10.1371/journal.pntd.0004389

**Published:** 2016-03-17

**Authors:** Kate E. Mounsey, Charlotte Bernigaud, Olivier Chosidow, James S. McCarthy

**Affiliations:** 1 Inflammation & Healing Research Cluster, School of Health and Sport Sciences, University of the Sunshine Coast, Maroochydore, Queensland, Australia; 2 Infectious Diseases & Immunology Division, QIMR Berghofer Medical Research Institute, Herston, Queensland, Australia; 3 Dermatology Department, Henri Mondor Hospital, AP-HP, UPEC, Créteil, France; 4 Université Paris-est Créteil Val de Marne, Créteil, France; 5 School of Medicine, University of Queensland, Herston, Queensland, Australia; Hitit University, Faculty of Medicine, TURKEY

## Scabies: An Underappreciated Global Health Problem

The recent addition of scabies to the World Health Organization’s list of neglected tropical diseases (NTDs) represents an important milestone in the understanding of the burden of disease that this infection imposes [1]. Finally, this most neglected of the neglected diseases may start to get the attention it deserves among global health policy makers and donors. It may also facilitate progress towards a sustainable approach to scabies control worldwide, as previously articulated by Engelman and colleagues [2].

In the 2010 Global Burden of Disease (GBD) study, scabies was among the 50 most common infectious diseases worldwide, with a point prevalence of around 100 million [3], although the precision of this estimate is hard to gauge because of a lack of quality prevalence studies [4]. In terms of morbidity, at 1.5 million disability-adjusted life years, scabies ranks higher than several other important NTDs, including dengue (0.83 million), onchocerciasis (0.43 million), and trypanosomiasis (0.56 million) [5]. It should be emphasised that this figure relates to the impact of scabies alone, with direct effects including itch, subsequent loss of sleep, school and work absences, and psychological distress. If one also considers the complications of scabies, including bacterial skin infection, post-streptococcal glomerulonephritis, and, potentially, rheumatic fever [2], the true global health burden attributed to this tiny ectoparasitic mite is much larger [4]. While frequently associated with poverty and overcrowding, scabies epidemics also remain problematic in developed countries. Why, then, does scabies continue to be relegated to the “nuisance” category, when other diseases attract considerably more research effort and funding?

## Inadequacy of Current Treatments for Scabies

The limited treatment options currently available for scabies are inadequate to tackle this global problem. Alternative approaches, such as immunotherapy [6], vaccination [7], or directly targeting mite molecules [8], have been proposed, but given the low research base and lack of interest from pharmaceutical companies, prospects for new drug development are dim. Acaricides that should be of historical interest, such as precipitated sulfur (messy and malodorous) and benzyl benzoate (highly irritating), remain the only affordable option in many developing countries [9]. 5% permethrin is safe and well tolerated, but prohibitively expensive in many countries—in the United States, a single application costs in excess of US$50. Furthermore, despite being deemed the most effective treatment for scabies in systematic reviews [10], clinical trial outcomes may not necessarily translate to the community, where poor adherence with topical regimens is a key determinant of scabies treatment failure. Although there are no publications confirming permethrin resistance in human scabies, anecdotal reports are increasingly common, and a laboratory population of *Sarcoptes scabiei* var. *canis* is highly resistant [11]. While permethrin treatment is relatively straightforward (apply the cream from head to toe, leave for at least 8 hours, and rinse off), this is impractical for community mass drug administration (MDA), and indeed, recent studies show limited sustainability of interventions in which treatment was not directly observed [12]. A qualitative study revealed barriers to appropriate use in scabies-endemic communities in northern Australia, including a lack of privacy to undertake whole-body application, insufficient facilities to rinse the cream off, and discomfort using the cream in tropical environments [13]. Similar sentiments regarding cumbersome application have been echoed by health practitioners in aged care facilities, in addition to the reluctance with administering full-body applications to mentally or physically disabled patients [14].

## Oral Ivermectin for Scabies

These issues with topical treatment adherence meant that the addition of oral ivermectin to the scabies arsenal in the mid-1990s was greeted with optimism [15]. Twenty years later, uptake of ivermectin for scabies has been relatively slow, with the primary indication being for institutional scabies outbreaks and for the treatment of severe crusted scabies, for which it has been mainly used off-label. Ivermectin is available at relatively low cost or has been provided free or heavily subsidized by manufacturers for use in large control programmes. For the treatment of ordinary scabies, it is only licenced in a few countries; in Australia and New Zealand, it is only registered as a second-line treatment where failure with topical creams has been observed, and in France, it is used as a single administration [16]. Meta-analyses [10] have shown that single-dose oral ivermectin is inferior to single-dose permethrin, meaning that two doses are required for maximal efficacy. This likely relates to its short plasma half-life in humans, with no apparent residual activity against eggs that may hatch after application over the 14-day mite life cycle. This is not ideal for MDA, in which administration of a second dose that is separated by several days is logistically problematic [17]. Where two doses were given, follow-up was frequent, and community visitors were continuously screened and treated, ivermectin MDA was more successful [18]. Conversely, recent efforts in Australian indigenous communities with single-dose ivermectin failed to show sustained reductions in scabies prevalence [19].

A major limitation to the use of ivermectin for scabies is its incompletely documented safety profile in key groups. Although millions of doses have been administered for onchocerciasis, with few well-documented serious adverse effects outside *Loa loa*-endemic areas, there remain concerns regarding its safety in children weighing less than 15 kg and in pregnancy and breastfeeding, although no increased risk has been found in cases of inadvertent exposure in these groups [20,21]. Overwhelmingly, scabies is a disease of the very young. Surveys of clinic attendances in Australian Aboriginal communities show that most presentations occurred in the population under 2 years of age [22], with this age distribution supported by a recent systematic review of scabies prevalence globally [4]. Whereas most literature recommends against administering ivermectin to children under 15 kg (approx. 3 ½ years, according to WHO growth charts), the current Australian package and MDA protocols expand this to children under 5, regardless of weight, which excludes a very large portion of the population at risk for scabies. This, combined with the requirement for pregnancy testing or exclusion of potentially pregnant women, severely limits the utility of ivermectin in community MDAs, with these groups instead receiving conventional topical treatment and the compliance problems they bring, which may partially explain why recent outcomes have been less than desired. Notably, topical 0.5% ivermectin has been approved in the US for the treatment of head lice in infants over 6 months of age, although bioavailability is much lower than that of oral administration [23]. Oral doses of up to 0.4 mg/kg have been administered to children as young as 2 in head lice trials [24]. A 1% topical ivermectin treatment was also recently approved by the US Food and Drug Administration (FDA) for the treatment of rosacea (presumably with activity against *Demodex* mites) [25,26], which is not explicitly contraindicated in pregnancy or breastfeeding, but rather, treatment may be warranted if benefits to the mother are perceived to outweigh the risk [27].

Another lingering concern with ivermectin is documentation of treatment failure despite multiple doses and in vitro evidence of resistance [28]. This was supported by observations of increasing in vitro survival times over the course of ivermectin treatment, suggesting that selection for resistant mites could occur relatively quickly [29]. The consequence of this is that ivermectin monotherapy is not recommended for cases of crusted scabies, with concomitant therapy with a topical acaricide such as permethrin and keratolytic supplementation required [30]. Observations of resistance in scabies may have implications for the more widespread use of oral or topical ivermectin for head lice and rosacea [26,31]. For crusted scabies, it is possible that the emergence of resistance in these cases may relate to low drug penetration and suboptimal mite exposure in hyperkeratotic areas of skin. However, remarkably little research has been undertaken on the distribution and retention of ivermectin in human skin. In one study, considerable variation in skin ivermectin concentration was reported and related to sebum levels. The authors contended that this could be a factor in determining clinical efficacy for scabies [32]. Concerns have been raised regarding the possibility of reduced activity of ivermectin in patients with xerotic skin, such as the elderly, which may explain treatment failures despite multiple doses in elderly patients [33]. Notably, the dose selection of 200 μg/kg is largely based on its potent activity against nematodes at this concentration, and no formal dose-finding studies have been undertaken for scabies, with some studies reporting reduced efficacy (<70%) at concentrations below 200 μg/kg [34–36], suggesting this dose may be around the minimum threshold of mite toxicity.

Looking at the prospects for scabies drug development, the target product profile for any new drug must be considered carefully. Of foremost importance is the preference for an oral treatment, ideally effective as a single dose for utility in the MDA setting. Moxidectin is a second-generation macrocyclic lactone, related to ivermectin but with critical pharmacokinetic differences. Currently under development as an alternative treatment for onchocerciasis, moxidectin also offers promise for human scabies. The significant advantage of moxidectin lies in its higher lipophilicity, leading to superior bioavailability (half-life >20 days versus 14 hours for ivermectin, [37]), and superior distribution and retention in tissue compared to ivermectin. When it comes to scabies, this factor could be a game changer—if the drug is retained at therapeutic concentrations in the skin through the 14-day scabies life cycle, a single-dose regimen may be possible.

## New Hope with Moxidectin?

Moxidectin is well established in veterinary practice to treat a range of parasites, including sarcoptic mange. This provides a solid foundation for considering its potential translation to human scabies. Some studies show excellent efficacy as a single 0.2 mg/kg dose, with 100% cure at day 14 in cattle [38], whereas sheep required two 0.2 mg/kg doses to achieve cure [39,40], with a single dose reducing mites by 75%–92%. When higher concentrations (1 mg/kg) of a long-acting formulation were used, 100% efficacy was achieved in a single dose [41]. Importantly, both 0.2 mg/kg and 1 mg/kg single-doses prevented reinfection from untreated animals for 25 and 54 days, respectively [41,42]. Differences in observed clinical efficacy may relate to the severity of infestation or pharmacokinetic differences between different species. Differences are also evident between injectable, oral tablet, and liquid formulations, so determining this in human pharmacokinetic profiling, including skin levels, would be ideal, in addition to controlled dose-finding efficacy studies.

Another important consideration is any potential differences in toxicity between ivermectin and moxidectin in target parasites. It is well documented that certain species of arthropods have reduced sensitivity to moxidectin compared to ivermectin. For example, the *Anopheles gambiae* toxic dose for moxidectin is over 100-fold higher than ivermectin [43], indicating that it may be unsuitable for use as an adjunct malaria control agent. This is especially important given the aforementioned issues with distribution and retention of the drug in the skin at therapeutic concentrations for sufficient periods. Preliminary studies in a porcine model of scabies are encouraging in this respect, with a single 0.3 mg/kg dose of moxidectin achieving high clinical efficacy with prolonged retention in skin [44].

Moxidectin is currently under consideration for regulatory submission for the treatment of onchocerciasis in humans. If successful, this would facilitate its development for scabies and other indications for which a long-acting macrocyclic lactone may be more effective than ivermectin. Early dose-escalation studies demonstrated that moxidectin is well tolerated in a dose range of 3–36 mg (up to ~0.6 mg/kg) [45]. Such doses would likely attain skin levels in therapeutic range for scabies. Limited studies have been done in lactating women, with a relative infant dose of 8.7% via breast milk—higher than ivermectin, but arguably within levels considered safe [46]. Phase II and III studies with moxidectin have now been completed for onchocerciasis [47], with promising results in regards to both efficacy and safety compared to ivermectin. However, as these trials have been conducted at lower concentrations than what may be required for effective treatment of scabies, it would be appropriate to assess its bioavailability and skin concentrations over the course of the mite life cycle. Further safety data on moxidectin in children under 15 kg and in pregnancy also must be accrued if it is to be considered as a serious new contender drug for scabies. In vitro and animal studies are promising in this respect, as they suggest that moxidectin is a poorer substrate for P-glycoproteins than ivermectin, and high-dose moxidectin can be administered to P-glycoprotein–deficient, ivermectin-sensitive dogs, with little evidence of toxicity [37]. This suggests that the risk for central nervous system toxicity associated with blood–brain barrier underdevelopment may be reduced with moxidectin, particularly if mutations in the human *MDR1A* gene are suspected to be associated with ivermectin severe adverse events [48]. Although in studies of onchocerciasis moxidectin was associated with an increased proportion of mild or moderate Mazzotti reactions (pruitis, rash, decreased blood pressure) [47], presumably related to its more potent microfilaricidal activity, these did not preclude further trials, and there has only been one report of a Mazzotti-type reaction in ivermectin-treated severe crusted scabies [49].

Clinical trials for assessing acaricidal agents are problematic. Meta-analysis indicates that few published studies pass muster, with significant heterogeneity in study designs evident [10]. Any scabies treatment protocol must not only consider the patient but all potential contacts. Current diagnostic methods for scabies are inadequate, and as such, diagnosis mostly relies on clinical presentation, which can be subjective even when well-defined clinical algorithms are employed. Human challenge trials for scabies would be logistically difficult and ethically problematic, given the long incubation period and potential to spread to personal contacts. Most scabies treatment evaluations have been undertaken in endemic communities, where it can be difficult to conduct adequately powered, well-controlled studies because of the inherent requirement to treat everyone in the community, both from an ethical perspective and to reduce confounding effects of reinfestation from untreated groups.

Given these significant challenges to executing properly powered efficacy studies in human populations and the very limited resources and funding available, a combination approach of conducting initial, preclinical or Phase I efficacy and dose-finding studies using an animal model—to complement the required pharmacokinetic and safety studies in humans—represents a rational strategy prior to conducting larger and more expensive Phase II and III trials in humans. Indeed, under the underutilised FDA “Animal Rule”[50], animal surrogates may be deemed acceptable when human challenge studies are not ethical or feasible. The recent development of a porcine model for human scabies [51] holds significant potential as a tool for scabies drug development, as pigs develop similar clinical responses to *S*. *scabiei* infestation and have similar skin physiology. Studies conducted to date suggest similar moxidectin pharmacokinetic profiles in pigs and humans [52], such that results obtained from porcine studies can be expected to inform dose selection and regimen in humans. Critically, in pigs, the opportunity exists to closely control infestation and undertake detailed clinical monitoring beyond that which could be performed in human participants.

## Final Remarks

With increasing recognition of scabies as a global health problem, improved control of scabies in endemic communities is achievable, pending support from donors and funding agencies and the availability of new treatments that are more amenable for use in MDA. Moxidectin is very promising, given its proven clinical efficacy against mange in animals, existing safety data in humans, and pharmacokinetic properties that may make it suitable for a single-dose regimen. However, due attention must firstly be paid to key development considerations, including comparative clinical efficacy, dose optimisation, epidermal and systemic pharmacokinetic and pharmacodynamic profile, and the acaricidal sensitivity threshold of *S*. *scabiei* to moxidectin. Finally, more data on the safety of moxidectin in young children and in pregnancy, and breastfeeding must be accumulated if this drug is to proceed as a genuine new candidate for the sustainable control of scabies.
